# Malnutrition, Sarcopenia, and Malnutrition-Sarcopenia Syndrome in Older Adults with COPD

**DOI:** 10.3390/nu14010044

**Published:** 2021-12-23

**Authors:** Aleksandra Kaluźniak-Szymanowska, Roma Krzymińska-Siemaszko, Ewa Deskur-Śmielecka, Marta Lewandowicz, Beata Kaczmarek, Katarzyna Wieczorowska-Tobis

**Affiliations:** Department of Palliative Medicine, Poznan University of Medical Sciences, 61-245 Poznan, Poland; krzyminskasiemaszko@ump.edu.pl (R.K.-S.); edeskur@ump.edu.pl (E.D.-Ś.); dietetyk.martalewandowicz@gmail.com (M.L.); bkaczmarek@ump.edu.pl (B.K.); kwt@tobis.pl (K.W.-T.)

**Keywords:** GLIM, EWGSOP2, diagnosis, screening, older individuals

## Abstract

Purpose: Chronic obstructive pulmonary disease (COPD) is the fourth leading cause of death in the world population. In addition to airflow obstruction, COPD is associated with multiple systemic manifestations, including impaired nutritional status or malnutrition and changes in body composition (low muscle mass, LMM). Poor nutritional status and sarcopenia in subjects with COPD leads to a worse prognosis and increases health-related costs. Data from previous studies indicate that 30–60% of subjects with COPD are malnourished, 20–40% have low muscle mass, and 15–21.6% have sarcopenia. This study aimed to assess the prevalence of malnutrition, sarcopenia, and malnutrition-sarcopenia syndrome in elderly subjects with COPD and investigate the relationship between COPD severity and these conditions.Patients and methods: A cross-sectional study involving 124 patients with stable COPD, aged ≥60, participating in a stationary pulmonary rehabilitation program. Nutritional status was assessed following the Global Leadership Initiative on Malnutrition (GLIM) criteria and sarcopenia with the European Working Group on Sarcopenia in Older People 2 (EWGSOP2) criteria. The results of pulmonary function tests and exercise capacity were obtained from the hospital database. Results: 22.6% of participants had malnutrition according to the GLIM criteria. Subjects with malnutrition had lower gait speed (*p* = 0.0112) and worse results of the Six Minute Walk Test. Sixteen participants (12.9%) had sarcopenia; 12 subjects with sarcopenia had concomitant malnutrition. The prevalence of severe and very severe obstruction (GOLD3/GOLD4) was 91.7%. It was significantly higher in patients with malnutrition-sarcopenia syndrome. Conclusions: Malnutrition was found in nearly one out of four subjects with COPD, while sarcopenia was one out of seven patients. About 10% of our study sample had malnutrition-sarcopenia syndrome. The prevalence of severe and very severe obstruction was significantly higher in patients with malnutrition-sarcopenia syndrome.

## 1. Introduction

Chronic obstructive pulmonary disease (COPD) is ranked highly among civilization diseases. Currently, it is the fourth leading cause of death in the world population, and it is predicted to be the third one in the nearest future [1]. Chronic obstructive pulmonary disease affects 12% of the world population [2]. However, the prevalence of COPD is 4times higher in subjects over 60 compared topeople younger than 50 (21.38% vs. 5.28%) [2].

COPD is an inflammatory condition [1]. In addition to airflow obstruction, it is associated with multiple systemic manifestations, which largely influence prognosis and treatment cost. Abnormal nutritional status and changes in body composition are among the most prevalent comorbidities in subjects with COPD, with a substantial, negative impact on prognosis (higher risk of COPD exacerbations, depression, or mortality) [3,4,5,6]. Data from previous studies indicate that 30–60%of subjects with COPD are malnourished [7,8,9], 20–40% have low muscle mass [3,4], and 15–21.6% have sarcopenia [10,11]. Under physiological conditions, an adult person expends 36–72 calories daily for breathing. Subjects with COPD and severe obstruction may expend even ten-fold more for this process. The enhanced calorie demand is not always compensated with diet [12]. Both malnutrition and sarcopenia negatively influence the course of COPD: they impair exercise tolerance, increase the risk of hospitalization, and decrease quality of life [4,10,12].Concomitance of malnutrition and sarcopenia (malnutrition-sarcopenia syndrome, MSS) increases the risk of death to a higher degree than malnutrition or sarcopenia alone [13]. Because of age-associated changes, elderly individuals are particularly prone to such complications [14].

A nutritional intervention (i.e., higher protein supply) aimed to increase muscle mass may favorably influence the rehabilitation process in undernourished elderly subjects with COPD, improving respiratory muscle force, physical capacity, overall health condition, and quality of life [6,15,16]. Such interventions must be preceded by proper diagnostics, i.e., assessment of nutritional status. According to the most recent diagnostic criteria formulated by the Global Leadership Initiative on Malnutrition (GLIM), malnutrition is diagnosed in an individual with a positive result of a screening test and at least one phenotypic (unintentional weight loss, low muscle mass, low body mass index) and at least one etiologic criterion (reduced food intake or assimilation, disease burden/inflammatory condition) [17]. Sarcopenia may be diagnosed based on recommendations from the European Working Group on Sarcopenia in Older People 2 (EWGSOP2). These recommendations include three criteria: low muscle strength (identification of probable sarcopenia, allows for therapeutic intervention), low muscle mass (necessary to confirm the diagnosis of sarcopenia), and low physical performance (determination of the severity of sarcopenia) [18].

While it is true that the prevalence of sarcopenia or malnutrition in subjects with COPD has been investigated in many studies, there are only a few reports in which the most recent diagnostic criteria (GLIM and EWGSOP2) were applied [19,20,21,22,23]; none of the studies involved both conditions. The present analysis aimed to assess the prevalence of malnutrition, sarcopenia, and malnutrition-sarcopenia syndrome in elderly subjects with COPD and investigate the relationship between COPD severity and these conditions.

## 2. Materials and Methods

We performed a cross-sectional analysis of a group of patients with chronic stable COPD from the Pulmonary Rehabilitation Ward (Great Poland Centre of Pulmonology and Thoracic Surgery) between September 2019 and November 2020. All patients admitted to the pulmonary rehabilitation ward in this period who met the inclusion criteria were enrolled.Each subject gave written informed consent before the study, conducted under the Declaration of Helsinki. The study protocol was approved by the Bioethical Committee of the Poznan University Medical Sciences, Poland (approval No: 888/19).

The inclusion criteria were as follows:

- age 60 years and more,
- COPD diagnosed based on recommendations from the Global Initiative for Chronic Obstructive Lung Disease (GOLD) [1],
- good verbal communication, absence of cognitive impairment,
- absence of active inflammation
- informed, written consent to participate in the study.

The exclusion criteria involved:

- active malignancy,
- contraindications for the body composition analysis with a bioimpedance method (metal implants, implanted cardiac devices, oedemas).

All participants had the following parameters assessed: cognitive performance, malnutrition (following the GLIM criteria), and sarcopenia (according to the EWGSOP2 recommendations). Additionally, the results of pulmonary function tests and exercise tests were obtained from the hospital database.

### 2.1. Assessment of Cognitive Performance

All patients were screened with the Abbreviated Mental Test Score (AMTS) to exclude subjects with cognitive impairment [24]. The AMTS questionnaire contains ten items. One point is given for each correct answer. Subjects that scored ≥7 (which indicates an absence of significant cognitive impairment) were recruited to the study.

### 2.2. Assessment of Malnutrition

Malnutrition was diagnosed based on the GLIM criteria [17]. In the first phase, we used the Mini Nutritional Assessment-Short Form questionnaire (MNA-SF) as a screening tool for malnutrition [25]. A score below 12 (out of the maximum of 14) indicates the risk for malnutrition.

The diagnosis of malnutrition was confirmed if at least one phenotypic and at least one etiologic criterion was fulfilled, as recommended by the GLIM experts.

Phenotypic criteria:

(1) unintentional body weight loss: the loss of >5% habitual body mass within the past six months, or the loss of >10% in more than six months,
(2) low body mass index (BMI): <20 kg/m^2^ in subjects below 70 years and <22 kg/m^2^ in individuals 70 years or older,
(3) low muscle mass (LMM): muscle mass was assessed based on the measurement of appendicular lean mass and calculation of the Appendicular Lean Mass (ALM) and an ALM index, which represents the ratio of ALM (kg) and square of height (m^2^). ALM index below the cut-off points for the Polish population (5.6 kg/m^2^ in women and 7.4 kg/m^2^ in men) [26] was indicative of low muscle mass (LMM).

The appendicular lean mass was measured with the electrical bioimpedance method (BIA) (InBody 120 analyzer, Biospace, Seoul, Korea).

Etiologic criteria:

(1) reduced food intake or assimilation was recognized in subjects declaring any reduction in food intake within the past three months in the MNA-SF questionnaire,
(2) the disease burden/inflammatory condition was recognized in all participants with a COPD diagnosis.

### 2.3. Assessment of Sarcopenia

Following the F-A-C-S (Find Cases-Assess-Confirm-Severity) algorithm [18], we used the SARC-F questionnaire as a screening tool for sarcopenia in the first phase of the diagnostic protocol [27]. The SARC-F questionnaire contains five items; the score of ≥4 indicates the risk of sarcopenia. Full diagnostics for sarcopenia wereperformed in all participants, regardless of the SARC-F results. This approach was based on the EWGSOP2 recommendations to perform such diagnostics in all subjects with a clinical suspicion of sarcopenia, and the assumption that all individuals with COPD have a clinical suspicion of sarcopenia.

#### 2.3.1. Assessment of Muscle Strength

The upper limb muscle strength was assessed based on a handgrip test with a hand dynamometer (Saehan, Changwon, Korea). The measurements were done with precision to 0.1 kg. The participants performed the handgrip test in a sitting position, with arms bent to 90 degrees in the elbow and arm joints. Each upper limb was tested twice. The mean of all measurements was used as a final result. The cut-off points for low muscle strength were following the EWGSOP2 recommendations (16 kg for women and 27 kg for men) [18].

The lower limb muscle strength was measured with a Chair Stand Test (CST) [28]. Participants were ordered to stand up repeatedly from a chair five times, with their arms crossed at the chest. Results < 15 s indicated low lower limb muscle strength.

Following the FACS algorithm, reduced muscle strength was diagnosed in individuals with decreased upper limb muscle strength and/or reduced lower limb muscle strength [18].

#### 2.3.2. Assessment of the Muscle Mass

The method adopted for muscle mass assessment was previously described in malnutrition diagnosis.

### 2.4. Pulmonary Function Tests—Spirometry

Information on the results of spirometry was obtained from the hospital database. Spirometry was performed with LUNGTEST 1000 device (MES, Cracow, Poland). All subjects enrolled in the study had chronic obstructive pulmonary disease diagnosed based on GOLD recommendations (post-bronchodilator forced expiratory volume in 1 s (FEV1)/forced vital capacity (FVC) ratio < 0.7) [1]. The severity of obstruction was staged based on FEV1:

- GOLD 1 (mild obstruction) FEV1 ≥ 80%
- GOLD 2 (moderate obstruction) FEV1 ≥ 50%
- GOLD 3 (severe obstruction) FEV1 ≥ 30%
- GOLD 4 (very severe obstruction) FEV1 < 30%

### 2.5. Exercise Capacity

Each participant performed the Six-Minute Walk Test (6MWT) to assess his/her physical capacity. Patients walked for six minutes in a straight corridor 30m long under the supervision of hospital physiotherapists, and the distance covered was measured. Blood pressure was taken, and blood oxygen saturation was measured with a pulse oximeter (Nonin Medical8500 A, Plymouth, MN, USA) directly before the test and after completing it. The 6MWT was finished earlier in case of dyspnea precluding its continuation, chest pain, severe lower limb pain, or cramps.

Although there are no cut-off points for the Six-Minute Walk Test, it is widely accepted [29,30,31,32] that a distancebelow 350 m is associated with a higher risk of COPD exacerbation, disease progression, and mortality.

### 2.6. Statistical Analysis

Qualitative data are presented as number (*n*) and percentage (%). For quantitative data, mean (M) and standard deviation (SD) are shown.

We used the following statistical tests: the Shapiro–Wilk test to assess the distribution of quantitative variables of normality; the Levene’s test to assess the equality of variances; the Student *t*-testto assess the difference between two groups for quantitative data showing normal distribution and homogeneity of variance; the Cochrane–Cox testto assess the difference between two groups for data with the normal distribution that do not show homogeneity of variance; the Mann–Whitney U testto assess the difference between two groups for quantitative data not showing normal distribution and discrete data; Kruskal-Wallis test to assess the difference between three groups, for quantitative data not showing normal distribution and discrete data. Categorical data were evaluated with the Pearson’s chi-squared test with Yates’s correction for continuity when at least one cellhad an expected count smaller than five (for 2 × 2 contingency tables) likelihood ratio chi-square tests (for contingency tables larger than 2 × 2). Fisher’s exact test was used for 2 × 2 contingency tables if the sample size was smaller than 40 and at least 1cell had an expected count smaller than 5. The *p*-value of less than 0.05 was considered significant. The statistical analysis was performed with STATISTICA 10 PL (StatSoft, Cracow, Poland).

## 3. Results

### 3.1. Characteristics of the Study Population

**Figure 1:**
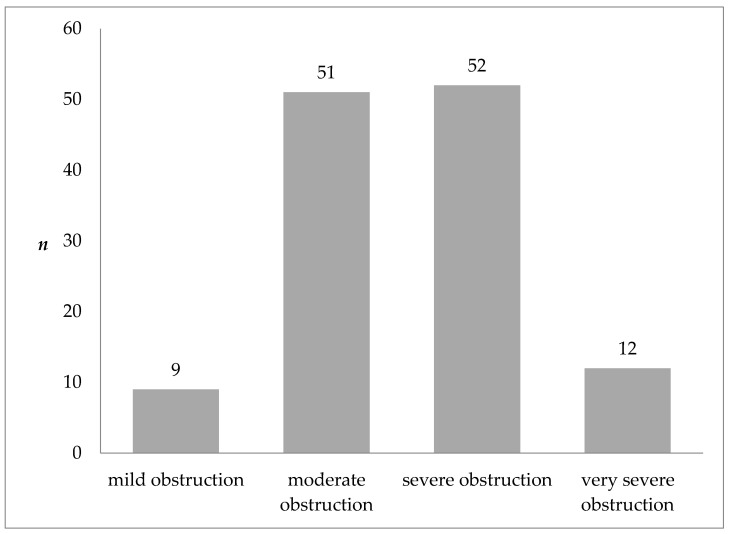
Severity of COPD according to the GOLD classification in the study population.

The study sample consisted of 124 patients of the pulmonary rehabilitation ward, aged at least 60 (mean age 69.4 ± 6.1 years). Forty percent of the study population were women. All subjects had COPD diagnosed based on the GOLD recommendations. Figure 1 shows the severity of airflow obstruction in the study population. As few persons had mild obstruction (*n* = 9) and very severe obstruction (*n* = 12), combined categories of mild to moderate obstruction (GOLD 1 + GOLD 2) and severe to very severe obstruction (GOLD 3 + GOLD 4) were used in further analysis.

### 3.2. Malnutrition

Thirty-eight subjects (30.6%) were at risk of malnutrition based on the MNA-SF score; the diagnosis of malnutrition was confirmed with the GLIM criteria in 28 patients (16 women and 12 men; 22.6% of the total study group). Table 1 shows the characteristics of subgroups with and without malnutrition according to the GLIM criteria. Subjects with malnutrition had lower BMI (*p* < 0.0000) and lower body composition parameters (BFM, SMM, PBF, FFM). There was a tendency towards higher upper and lower limb strength in patients with normal nutritional status (*p* = 0.0567). Malnourished participants had lower gait speed (*p* = 0.0112) and worse results forthe 6MWT (*p* = 0.0079). They also scored lower in the SARC-F questionnaire (*p* = 0.0336).

### 3.3. Sarcopenia and MSS

**Figure 2:**
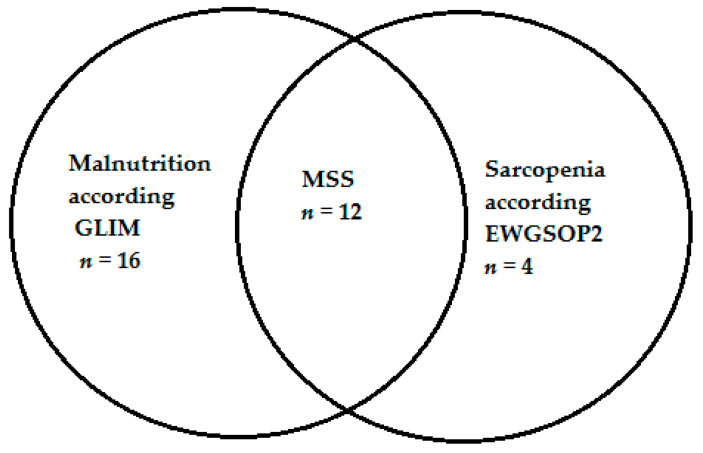
Number of subjects with malnutrition, sarcopenia and malnutrition-sarcopenia syndrome. Notes: GLIM, Global Leadership Initiative on Malnutrition; MSS, malnutrition-sarcopenia syndromeEWGSOP2, European Working Group on Sarcopenia in Older People 2.

Sarcopenia was diagnosed in 16 patients (12.9%); 12 of them had malnutrition concomitantly (malnutrition-sarcopenia syndrome, MSS) (Figure 2).

Table 2 shows characteristics of subgroups with and without sarcopenia. Individuals with sarcopenia had lower upper limb strength (*p* < 0.0001) and lower gait speed (*p* = 0.0395) than subjects without this condition. Significant differences in body composition were also observed. Patients with sarcopenia had lower body weight, BMI, BFM, SMM, FFM, FMM and PBF. Additionally, these subjects had worse results of the 6MWT (*p* = 0.0001) and had lower FEV1 (*p* = 0.0002), indicating more severe obstruction in comparison with participants without sarcopenia.

Malnutrition-sarcopenia syndrome was diagnosed in 1 out of 10 participants. Table 3 shows comparisons of patients with and without MSS. Subjects with concomitant malnutrition and sarcopenia had worse anthropometric parameters (body weight, BMI, BFM, SMM, FFM) and lower FEV1/FVC EX (*p* = 0.0396) and FEV1 (*p* = 0.0171). Moreover, subjects with MSS covered shorter distance during the 6MWT (*p* = 0.0002) as compared to individuals without the syndrome.

Table 4 presents comparisons between participants without malnutrition or sarcopenia, subjects with malnutrition or sarcopenia, and patients with malnutrition-sarcopenia syndrome. Individuals with MSS had significantly lower body weight compared to subgroup without malnutrition or sarcopenia (*p* = 0.0000) and subgroup with only one condition (*p* = 0.0480). The MSS subgroup had lower BMI than subjects without any condition (*p* = 0.0000) and patients with one condition (*p* = 0.0471).Participants with concomitant malnutrition and sarcopenia also had significantly lower muscle mass and lower body fat mass. Although the spirometry results did not differ between the subgroups, the prevalence of severe and very severe obstruction was higher in subjects with MSS. Patients with concomitant malnutrition and sarcopenia covered a shorter distance during the 6MWT than subjects without any condition (*p* = 0.0003) and individuals with malnutrition or sarcopenia alone (*p* = 0.0315).

## 4. Discussion

To the best of our knowledge, this is the first study to assess the prevalence of malnutrition, sarcopenia, and malnutrition-sarcopenia syndrome using the most recent diagnostic criteria (GLIM and EWGSOP2) in elderly subjects with COPD. Both malnutrition and sarcopenia are common in patients with COPD. Malnutrition may affect about 50% of elderly individuals with COPD [17], and sarcopenia may be present in one in four to one out of seven patients, particularly those with severe and very severe obstruction [18,19,20,21]. Catabolic processes are intensified both in COPD and aging, leading to the reduction of muscle mass. Muscle mass reduction is a diagnostic criterion for both malnutrition and sarcopenia. It is associated with more frequent exacerbations, more severe dyspnea, worse pulmonary function, and lower results in exercise tests [5]. It should be noted that COPD itself fulfills one of the etiologic GLIM criteria for malnutrition (disease burden/inflammatory condition). Therefore, all elderly subjects with COPD should be screened for malnutrition and sarcopenia. An early therapeutic intervention, including nutritional support, may positively influence the disease and prognosis.

Almost one of four participants had malnutrition in our study population, and one out of seven had sarcopenia. Sarcopenia was much more prevalent in patients with severe and very severe airflow obstruction. It should be emphasized that both conditions were diagnosed in nearly 10%of subjects. Malnutrition may lead to sarcopenia due to inadequate calorie and protein intake necessary for maintaining muscle mass. Sarcopenia, in turn, may exacerbate malnutrition by reducing mobility necessary for the everyday preparation of meals and food shopping [33]. Consequences of malnutrition and sarcopenia are largely overlapping [23]. The vast majority (92%) of patients with sarcopenia in our analysis had severe or very severe airflow obstruction, while only 37% of subjects with malnutrition alone were classified as GOLD 3/GOLD 4.

The prevalence of malnutrition and sarcopenia in subjects with COPD has been assessed in many studies. However, these studies used various screening tools and not up-to-date diagnostic criteria (e.g., the European Society for Clinical Nutrition and Metabolism (ESPEN) criteria for the diagnosis of malnutrition from 2015 or the older EWGSOP criteria for sarcopenia from 2010), which makes a direct comparison difficult [3,8,11,34,35,36,37]. We used the most recent guidelines for diagnostics of both conditions, which were first presented during the European Union Geriatric Medicine Society (EUGMS) congress in Berlin in October 2018. We found only two analyses using these up-to-date malnutrition diagnostic criteria [17]. However, these studies were not restricted to elderly subjects. Moreover, none of these studies investigated the concomitant presence of malnutrition and sarcopenia in patients with COPD. Dávalos-Yerovi et al. [17] assessed the prevalence of malnutrition in a group of 167 patients with COPD (aged 66.5 ± 9.0 years) addressed for pulmonary rehabilitation. The risk of malnutrition (based on the MNA-SF score) was diagnosed in 49% of participants. The diagnosis of malnutrition was further confirmed with the GLIM criteria in 45% of the study population. Additionally, Dávalos-Yerovi et al. observed a higher risk of hospitalization and death in malnourished patients. The high prevalence of malnutrition (double that observed in our study) may be explained by the fact that more than 70% of patients involved in the analysis had severe to very severe obstruction. The percentage of patients classified as GOLD 3 or 4 in our analysis was lower (51.6%). Araujo et al. included 241 subjects with COPD (aged 68.3 ± 10.2 years). The prevalence of malnutrition according to the GLIM criteria was 47.8% in their study sample. However, the investigators did not perform screening tests in the first step of the diagnostic procedure, whichcould explain such high percentage of malnutrition [38].

We found four studies assessing the prevalence of sarcopenia diagnosed based on the EWGSOP2 criteria in patients with COPD, including two papers concerning elderly subjects [18,19,20,21].Tsekoura et al. [18] analyzed a group of 69 elderly patients with COPD (mean age 71.33 ± 7.48 years). They diagnosed sarcopenia in 17 participants (24.6%). Marco et al. [19] studied 79 community-dwelling COPD rehabilitation patients (mean age 67.5 ± 7.1 years). They found sarcopenia in 10 persons (12.7%), which is very similar to the prevalence of sarcopenia in our study population (12.9%). The remaining two studies were not restricted to elderly subjects. Araújo et al. [20] included 208 patients (mean age 67.6 ± 10.1 years) hospitalized for COPD exacerbation. They diagnosed sarcopenia in 16.3% of participants. They also observed a correlation between COPD severity (GOLD 3 and 4), malnutrition, and sarcopenia, in accordance with our findings. A similar prevalence of sarcopenia (16.8%) was reported byDávalos-Yerovi et al. [21], in their paper concerning 95 patients with stable COPD (mean age 66.6 ± 9 years), published in 2019. They observed an increased risk of death and hospitalization in two years. The prevalence of sarcopenia in both discussed analyses [20,21] was similar to our results. It is noteworthy that 75% (*n* = 12) of patients with sarcopenia in our study had concomitant malnutrition. In addition to an association between sarcopenia and COPD severity, we observed a relationship between sarcopenia and the results of pulmonary function tests and 6MWT tests.

Lengelé et al. [39] have demonstrated in their recent analysis, involving 418 community-dwelling elderly subjects, that the GLIM criteria, but not the MNA-SF score, predict sarcopenia in five years. They emphasized that impaired nutritional status, resulting from inadequate protein intake, may significantly contribute to sarcopenia. These observations are in accordance with our findings. Moreover, Lengelé et al. noticed that among 59 individuals diagnosed as malnourished based solely on etiologic and phenotypic GLIM criteria, as many as 32 subjects (54%) had normal nutritional status based on the MNA-SF score. The results of our previous work also demonstrated just fair diagnostics performance of the MNA-SF questionnaire as a screening tool in the GLIM criteria. Thus, we suggested using a clinical suspicion of malnutrition interchangeably with a screening tool in the first step of the GLIM algorithm [40].

It should be emphasized that overlooking of malnutrition may lead to sarcopenia, particularly in elderly subjects. Concomitance of these conditions increases the risk of death, as evidenced by Hu et al. [11] in the analysis of 453 hospitalized elderly patients (mean age 79.0 ± 7.8 years). They observed a twofold higher risk of death in subjects with MSS as compared to patients with malnutrition or sarcopenia alone (HR for MSS: 4.78; HR for malnutrition: 2.62; HR sarcopenia: 1.66) [11].

Despite the well-known adverse consequences of malnutrition and sarcopenia and their high prevalence in elderly subjects with COPD, both conditions are rarely diagnosed in such patients. Lack of diagnosis precludes implementation of therapeutic intervention. Based on our results, we suggest screening for both malnutrition and sarcopenia in all elderly subjects with COPD, regardless of its severity. Such an approach would necessitate the involvement of a dietician and a physiotherapist and should positively influence prognosis in such patients.

Our study has some limitations. First, the study group consisted of patients involved in pulmonary rehabilitation, so their physical condition must have been relatively good—to allow for such activity. Therefore, our results are not representative of the total COPD population. Secondly, we assessed the GLIM etiologic criterion concerning reduced food intake or assimilation using the subjective answers to the MNA-SF questionnaire items. Although subjects enrolled in our study had no cognitive impairment, determining if one’s food intake declined over the past three months due to loss of appetite, digestive problems, and chewing or swallowing difficulties (the MNA-SF item) might have been difficult for some of them. Moreover, the lack of multivariate analyses is a limitation of our study.

The strong point of our study is that this is the first analysis of the prevalence of both malnutrition and sarcopenia in elderly patients with COPD, in which the most recent diagnostic criteria have been used. It should be emphasized that elderly patients with COPD are at particular risk for both conditions.

## 5. Conclusions

Malnutrition according to the GLIM criteria was found in nearly one out of four subjects with COPD, while sarcopenia according to the EWGSOP2 in one out of seven patients. About ten percent of our study sample had malnutrition-sarcopenia syndrome. The prevalence of severe and very severe obstruction was significantly higher in patients with malnutrition-sarcopenia syndrome. Subjects with the MSS had also worse results of the Six Minute Walk Test, which is a poor prognostic factor in COPD.

## Figures and Tables

**Table 1:** Characteristics of subgroups with and without malnutrition according to the GLIM criteria.

	Without Malnutrition *n* = 96	Malnutrition *n* = 28	*p*-Value
Age (years)	69.4 ± 6.1	69.4 ± 6.0	0.9332 ^a^
AMTS	9.6 ± 0.7	9.6 ± 0.6	0.5590 ^a^
Height (cm)	167.5 ± 8.7	162.8 ± 10.2	0.0111 ^a^
Body weight (kg)	82.9 ± 18.3	61.0 ± 19.2	0.0000 ^a^
BMI (kg/m^2^)	29.5 ± 6.0	22.8 ± 6.0	0.0000 ^a^
BFM (kg)	28.7 ± 12.2	17.6 ± 11.1	0.0000 ^a^
SMM (kg)	30.0 ± 6.2	23.4 ± 6.3	0.0000 ^a^
PBF (%)	33.6 ± 9.4	26.9 ± 10.0	0.0019 ^a^
FFM (kg)	22.4 ± 5.0	16.9 ± 5.1	0.0000 ^a^
MNA-SF	13.1 ± 1.1	8.6 ± 2.6	0.0000 ^a^
Low ALM index	10 (10.4)	18 (64.3)	0.0000 ^c^
Low upper limb strength	32 (33.3)	14 (50.0)	0.1082 ^c^
Chair Stand Test(s)	14.2 ± 5.7	17.1 ± 7.9	0.0567 ^a^
4-m usual walking speed test (m/s)	0.9 ± 0.2	0.8 ± 0.2	0.0112 ^b^
SARC-F	2.0 ± 1.6	3.0 ± 2.0	0.0336 ^a^
Risk of sarcopenia according SARC-F	18 (18.8)	11 (39.3)	0.0239 ^c^
FEV1/FVC EX	51.5 ± 13.1	49.3 ± 10.9	0.2781 ^a^
FEV1	51.0 ± 18.7	49.3 ± 19.5	0.5620 ^a^
GOLD 1 + GOLD 2	49 (81.7)	11 (18.3)	0.2734 ^c^
GOLD 3 + GOLD 4	47 (73.4)	17 (26.6)	
6 MWT (m)	372.2 ± 118.3	281.0 ± 143.4	0.0079 ^a^

Notes: Values are presented as numbers (%) or mean ± standard deviation for descriptive analyses. AMTS, Abbreviated Mental Test Score; BMI, body mass index; BFM, body fat mass; SMM, skeletal muscle mass; PBF, percent body fat; FFM, free fat mass; MNA-SF, mini nutritional assessment—short form; ALM index, appendicular lean mass index; FEV1/FVC EX, forced expiratory volume in 1 s/forced vital capacity; FEV1, forced expiratory volume in 1 s; GOLD, Global Initiative for Chronic Obstructive Lung Disease; 6MWT, sixminute walk test. ^a^ Mann–Whitney test, ^b^ Student T-test, ^c^ Chi-square test.

**Table 2:** Characteristics of subgroups with and without sarcopenia according to the EWGSOP2 criteria.

	Without Sarcopenia *n* = 108	Sarcopenia *n* = 16	*p*-Value
Age (years)	69.2 ± 5.9	70.4 ± 7.5	0.6542 ^a^
AMTS	9.6 ± 0.7	9.8 ± 0.6	0.2243 ^a^
Height (cm)	166.8 ± 9.3	163.7 ± 8.8	0.0722 ^a^
Body weight (kg)	81.3 ± 19.5	55.2 ± 11.2	0.0000 ^a^
BMI (kg/m^2^)	29.1 ± 6.3	20.6 ± 3.6	0.0000 ^a^
BFM (kg)	27.9 ± 12.6	14.4 ± 6.7	0.0000 ^a^
SMM (kg)	29.5 ± 6.5	21.9 ± 4.0	<0.0001 ^b^
PBF (%)	33.2 ± 9.8	25.1 ± 8.2	0.0020 ^a^
FFM (kg)	21.9 ± 5.4	16.0 ± 3.5	<0.0001 ^b^
MNA-SF	12.5 ± 2.1	9.3 ± 3.1	0.0000 ^a^
Low ALM index	12 (11.1)	16 (100)	0.0000 ^c^
Low upper limb strength	32 (29.6)	14 (87.5)	<0.0001 ^c^
Chair Stand Test(s)	14.4 ± 6.0	18.6 ± 7.9	0.0536 ^a^
4-m usual walking speed test (m/s)	0.9 ± 0.2	0.8 ± 0.2	0.0395 ^b^
SARC-F	2.1 ± 1.7	3.0 ± 1.9	0.1819 ^a^
Risk of sarcopenia according EWGSOP2	24 (22.2)	5 (31.3)	0.4391 ^c^
FEV1/FVC EX	52.0 ± 12.8	44.1 ± 8.8	0.0396 ^a^
FEV1	52.5 ± 18.8	38.3 ± 14.0	0.0171 ^a^
GOLD 1 + GOLD 2	58 (53.7)	2 (12.5)	0.0011 ^c^
GOLD 3 + GOLD 4	50 (46.3)	14 (87.5)	
6 MWT (m)	371.3 ± 119.7	220 ± 118.7	0.0002 ^a^

Notes: Values are presented as numbers (%) or mean ± standard deviation for descriptive analyses. AMTS, Abbreviated Mental Test Score; BMI, body mass index; BFM, body fat mass; SMM, skeletal muscle mass; PBF, percent body fat; FFM, free fat mass; MNA-SF, mini nutritional assessment—short form; ALM index, appendicular lean mass index; FEV1/FVC EX, forced expiratory volume in 1 s/forced vital capacity; FEV1, forced expiratory volume in 1 s; GOLD, Global Initiative for Chronic Obstructive Lung Disease; 6MWT, sixminute walk test. ^a^ Mann–Whitney test. ^b^ Student T-test, ^c^ Chi-square test.

**Table 3:** Characteristics of subgroups with and without malnutrition-sarcopenia syndrome.

	Without MSS *n* = 112	MSS *n* = 12	*p*-Value
Age (years)	69.2 ± 5.9	70.6 ± 7.3	0.6354 ^a^
AMTS	9.6 ± 0.7	9.8 ± 0.6	0.2243 ^a^
Height (cm)	166.9 ± 9.2	162.0 ± 9.5	0.0722 ^a^
Body weight (kg)	80.9 ± 19.4	50.9 ± 8.1	0.0000 ^a^
BMI (kg/m^2^)	28.9 ± 6.3	19.5 ± 3.1	0.0000 ^a^
BFM (kg)	27.7 ± 12.5	12.3 ± 5.3	0.0000 ^a^
SMM (kg)	29.4 ± 6.5	20.5 ± 3.3	0.0000 ^b^
PBF (%)	33.0 ± 9.7	23.5 ± 8.3	0.0020 ^a^
FFM (kg)	21.9 ± 5.3	14.7 ± 3.0	0.0000 ^b^
MNA-SF	12.5 ± 2.0	8.2 ± 2.6	0.0000 ^a^
Low ALM index	16 (14.3)	12 (100)	0.0000 ^c^
Low upper limb strength	35 (31.3)	11 (91.7)	0.0000 ^c^
Chair Stand Test(s)	14.4 ± 6.0	18.6 ± 7.9	0.0536 ^a^
4-m usual walking speed test (m/s)	0.9 ± 0.2	0.7 ± 0.2	0.0159 ^b^
SARC-F	2.1 ± 1.6	3.1 ± 2.2	0.1819 ^a^
Risk of sarcopenia according SARC-F	24 (21.4)	5 (41.7)	0.1377 ^c^
FEV1/FVC EX	51.7 ± 12.8	44.7 ± 8.9	0.0396 ^a^
FEV1	51.9 ± 18.9	39.1 ± 14.1	0.0171 ^a^
GOLD 1 + GOLD 2	59 (52.7)	1 (8.3)	0.0016 ^c^
GOLD 3 + GOLD 4	53 (47.3)	11 (91.7)	
6 MWT (m)	368.5 ± 119.7	204.6 ± 122.4	0.0002 ^a^

Notes: Values are presented as numbers (%) or mean ± standard deviation for descriptive analyses. MSS, malnutrition-sarcopenia syndrome; AMTS, Abbreviated Mental Test Score; BMI, body mass index; BFM, body fat mass; SMM, skeletal muscle mass; PBF, percent body fat; FFM, free fat mass; MNA-SF, mini nutritional assessment—short form; ALM index, appendicular lean mass index; FEV1/FVC EX, forced expiratory volume in 1 s/forced vital capacity; FEV1, forced expiratory volume in 1 s; GOLD, Global Initiative for Chronic Obstructive Lung Disease; 6MWT, sixminute walk test. ^a^ Mann–Whitney test, ^b^ Student-T test, ^c^ Chi-square test.

**Table 4:** Comparison between participants without malnutrition or sarcopenia, subjects with malnutrition or sarcopenia, and patients with malnutrition-sarcopenia syndrome.

	Without Malnutrition or Sarcopenia ^a^ *n* = 92	Malnutrition or Sarcopenia ^b^ *n* = 20	Malnutrition-Sarcopenia Syndrome ^c^ *n* = 12	*p*-Value
Age (years)	69.4 ± 6.0	68.8 ± 5.8	70.6 ±7.3	NS
AMTS	9.6 ± 0.7	9.5 ± 0.6	9.8 ± 0.6	NS
Height (cm)	167.4 ± 8.9	164.5 ± 10.1	162.0 ± 9.5	NS
Body weight (kg)	83.6 ± 18.3	68.4 ± 19.7	50.9 ± 8.1	a vs. b *p* = 0.0054 ^d^ a vs. c *p* = 0.0000 ^d^ b vs. c *p* = 0.0480 ^d^
BMI (kg/m^2^)	29.8 ± 6.0	25.1 ± 6.0	19.5 ± 3.1	a vs. b *p* = 0.0083 ^d^ a vs. c *p* = 0.0000 ^d^ b vs. c *p* = 0.0471 ^d^
BFM (kg)	29.1 ± 12.3	21.4 ± 11.7	12.3 ± 5.3	a vs. b *p* = 0.0398 ^d^ a vs. c *p* < 0.0001 ^d^
SMM (kg)	30.2 ± 6.2	25.7 ± 6.5	20.5 ± 3.3	a vs. b *p* = 0.0117 ^d^ a vs. c *p* < 0.0001 ^d^
PBF (%)	33.8 ± 9.5	29.5 ± 9.9	23.5 ± 8.3	a vs. c *p* = 0.0027 ^d^
FFM (kg)	22.5 ± 5.1	18.8 ± 5.2	14.7 ± 3.0	a vs. b *p* = 0.0103 ^d^ a vs. c *p* < 0.0001 ^d^
MNA-SF	13.2 ± 1.1	9.7 ± 2.9	8.2 ± 2.6	a vs. b *p* = 0.0000 ^d^ a vs. c *p* = 0.0000 ^d^
Low ALM index	6 (6.5)	10 (50)	12 (100)	*p* = 0.0000 ^e^
Low upper limb strength	29 (31.5)	6 (30)	11 (91.7)	*p* = 0.0002 ^e^
Chair Stand Test(s)	14.2 ± 5.8	15.5 ± 7.2	18.6 ± 7.9	NS
4-m usual walking speed test (m/s)	0.9 ± 0.2	0.8 ± 0.2	0.7 ± 0.2	NS
SARC-F	2.0 ± 1.6	2.9 ± 1.7	3.1 ± 2.2	NS
Sarcopenia risk according to SARC-F	18 (19.6)	6 (30.0)	5 (41.7)	NS
FEV1/FVC EX	51.9 ± 13.2	50.6 ± 11.5	44.7 ± 8.9	NS
FEV1	51.7 ± 18.6	52.8 ± 20.5	39.1 ± 14.1	NS
GOLD 1 + GOLD 2	48 (52.2)	11 (55.0)	1 (8.3)	*p* = 0.0068 ^e^
GOLD 3 + GOLD 4	44 (47.8)	9 (45.0)	11 (91.7)	
6MWT (m)	375.6 ± 118.2	335.0 ± 124.5	204.6 ± 122.4	a vs. c *p* = 0.0003 ^d^ b vs. c *p* = 0.0315 ^d^

Notes: Values are presented as numbers (%) or mean ± standard deviation for descriptive analyses. NS, not significant; MSS, malnutrition-sarcopenia syndrome; AMTS, Abbreviated Mental Test Score; BMI, body mass index; BFM, body fat mass; SMM, skeletal muscle mass; PBF, percent body fat; FFM, free fat mass; MNA-SF, mini nutritional assessment—short form; ALM index, appendicular lean mass index; FEV1/FVC EX, forced expiratory volume in 1 s/forced vital capacity; FEV1, forced expiratory volume in 1 s; GOLD, Global Initiative for Chronic Obstructive Lung Disease; 6MWT, sixminute walk test. ^a^ participants without malnutrition or sarcopenia, ^b^ participantswithmalnutrition or sarcopenia, ^c^ participants with malnutrition-sarcopenia syndrome, ^d^ Kruskal–Wallis Test, ^e^ Chi-square test.

## Data Availability

All relevant data are within the manuscript and are openly available in the Zenodo repository (doi:10.5281/zenodo.5500015).
